# Effect of neonatal neuronal intensive care unit on neonatal encephalopathy

**DOI:** 10.1371/journal.pone.0261837

**Published:** 2021-12-31

**Authors:** Lu Lin, Weiqin Liu, Jing Mu, Enmei Zhan, Hong Wei, Siqi Hong, Ziyu Hua

**Affiliations:** 1 Department of Neonatology, Children’s Hospital of Chongqing Medical University, Chongqing, China; 2 Ministry of Education Key Laboratory of Child Development and Disorders, National Clinical Research Center for Child Health and Disorders, China International Science and Technology Cooperation Base of Child Development and Critical Disorders, Chongqing Key Laboratory of Child Health and Nutrition, Children’s Hospital of Chongqing Medical University, Chongqing, P.R China; 3 Department of Neurology, Children’s Hospital of Chongqing Medical University, Chongqing, China; Universita degli Studi di Napoli Federico II, ITALY

## Abstract

Prophylaxis of brain injury in newborns has been a main concern since the first neonatal neuronal intensive care unit (NNICU) was established in the world in 2008. The aim of this study was to outline and evaluate the unit’s development by analyzing the demographics of the patients, the services delivered, the short-term outcomes before and after the establishment of NNICU. During the two investigation periods, 384 newborns were diagnosed or suspected as “neonatal encephalopathy”, among which 185 patients admitted to NNICU between 2011.03.01 and 2012.09.30 before the establishment of NNICU were enrolled in the pre-NNICU group, another 199 neonates hospitalized during 2018.03.01 to 2019.09.30 were included in the post-NNICU group. Patients in the post-NNICU group were more likely to have seizures (*P* = 0.001), incomplete or absent primitive reflexes (*P* = 0.002), therapeutic hypothermia (*P*<0.001) and liquid control (*P*<0.001) in acute phase. Meanwhile, amplitude-integrated electro encephalogram (aEEG) monitoring (*P*<0.001) and cranial ultrasound (*P*<0.001) were more often used in NNICU. Both of the follow-up rate in brain MRI and the assessment of neurodevelopment at 3 months were higher in the post-NNICU group (*P*<0.001). In conclusion, the NNICU focused on the neonatal neurocritical care for the babies susceptible to NE with the guidance of evidence-based medicine, the establishment of NNICU is gradually improving and standardizing the neuroprotective therapy and clinical follow-up to improve neurodevelopmental prognosis of the NE patients in CHCMU.

## Introduction

Neonatal encephalopathy (NE) is a clinically defined syndrome of disturbed neurologic function in an infant born at or beyond 35 weeks of gestation, manifested by a subnormal level of consciousness or seizures, and often accompanied by difficulty with initiating and maintaining respiration and depression of muscle tone and primitive reflexes [1–3]. The outcomes following neonatal encephalopathy include death and neurological disabilities such as cerebral palsy, epilepsy and cognitive impairment [4, 5]. With the great advances in neonatal intensive care management, the mortality of preterm neonates has reduced, whereas neurodevelopmental morbidity persists at relatively high rates, causing greatly increased economic burden to the society. Therefore, the focus of neonatal medicine has now shifted to scrutinize the long-term outcomes of neonatal intensive care unit (NICU) graduates. Prophylaxis of brain injury in newborns is now a main concern.

In 2008, the first neonatal neuronal intensive care unit (NNICU) was operationalized at the university of California San Francisco (UCSF), then the other NICUs around world followed their lead [6–9]. There have been several prior publications describing the evolution of neurocritical units for neonates since 2008 [6, 9–11]. Glass H *et al*. reported the details of neurocritical care for neonates between July 2008 and June 2009 after the NNICU established at the UCSF. According to the study, specialized neurocritical care might improve neurodevelopmental outcomes for newborns, not merely survival, whereas it is a cross-sectional study, further longitudinal studies are needed. In 2014, Mulkey S *et al*. compared the change in neurologic care after the NNICU established in a tertiary care hospital in America, in that study, the period post-NNICU had a greater number of neurology consultations and better neuromonitoring, the establishment of NNICU could provide focused neurologic care to newborns. However, the demographic features and the clinical manifestations of the patients during the two periods were not described in that study, the neurodevelopmental outcomes were also not mentioned.

To better provide neurologically focused neonatal intensive care to a broad patient population, the first expert consensus on the establishment of NNICU was published in 2018 in China, and a NNICU was established at Children’s Hospital of Chongqing Medical University (CHCMU) at the same year, specialists in neonatology, neurology, neuroradiology, and neurodevelopmental care came together to conduct the multidisciplinary neonatal neurocritical care service. Therefore, this longitudinal study was designed and aimed to outline and evaluate the unit’s development by analyzing the demographics of the patients, the services delivered, the short-term outcomes before and after the establishment of NNICU.

## Patients and methods

Before NNICU establishment, there was no consensus on the strategies of preventing brain injury in China. Since the guideline for therapy of hypoxic ischemic encephalopathy (HIE) published in 2011 [12], preventing brain injury was considered more and more important.

In the department of neonatology at CHCMU, therapeutic hypothermia was used for asphyxia neonates since 2011, then other neuroprotective-related approaches such as amplitude-integrated electro encephalogram (aEEG) and neurodevelopment assessment were gradually introduced into this service during these years. Until several NNICUs were established in developed countries, the staffs in our department tried to develop a strategy for a dedicated service to provide specialized care for neonates with neurological conditions in 2017, then the specialists in neonatology, neurology, neuroradiology and neurodevelopmental care came together to accomplished it from 2018.

Therefore, in this retrospective analysis, the study period from 2011.03.01 to 2012.09.30 was the 18-months pre-NNICU and the period from 2018.03.01 to 2019.09.30 was the 18-months post-NNICU.

The medical records of neonates who were suspected neonatal encephalopathy or had primary medical diagnosis known to be associated with neurologic compromise during the study periods were collected. Patients were excluded if they had gestational age (GA) <35 weeks or hospitalized in CHCMU after the acute phase. In addition, those without complete general demographic records were also excluded. The clinical data were then recorded, including sex, gestational age, birth weight, risk factors, clinical manifestations and the result of amplitude-integrated electroencephalogram (aEEG) and neuroimaging (cranial ultrasonography, magnetic resonance imaging). Then, the neuroprotective therapy, the assessment of neurodevelopment and the short-term prognosis were also collected. The neurodevelopment was evaluated by experienced and certified examiners, neonatal behavioral neurological assessment (NBNA) or development quotient (DQ) were used for patients in the pre-NNICU group, whereas the test of infant motor performance (TIMP) was used for patients in the post-NNICU group. The short-term prognosis was defined as the results of the assessment of neurodevelopment for patients after birth until the follow-up at 3 months after birth.

We confirm adherence to ethical guidelines and indicate ethical approvals (IRB). Ethics approval for this study was obtained from the Institutional Review Board of the Children’s Hospital, Chongqing Medical University (Approval No. 2019–285). The need for written informed consent from the patients or their legal guardians was waived by the ethics committee because this was a retrospective study, and the data were collected and interpreted anonymously.

### Statistical analysis

All data were analyzed using *SPSS 22*.*0* software for windows. Measurement data were reported as the *mean* ± *standard deviation* or median (*interquartile range*). Count data were reported as numbers of cases. The *Kolmogorov-Smirnov* test for normality was conducted. The measurement data were analyzed using *t*-test if the data were normally distributed; otherwise, the *Mann-Whitney U* test was adopted. Count data were analyzed using the *Chi-squared* test. A multivariate analysis was applied to analyze the differential diagnosis. *P* value less than 0.05 were considered statistically significant.

## Results

During the two investigation periods, 384 newborns were diagnosed or suspected as “neonatal encephalopathy”, of which 185 patients admitted between 2011.03.01 and 2012.09.30, another 199 neonates hospitalized during 2018.03.01 to 2019.09.30. The demographic and risk factors comparison of them were shown in Table 1.

**Table 1:** Demographic features and risk factors of the neonates.

		Pre-NNICU (n = 185)	Post-NNICU (n = 199)	*χ* ^*2*^ */Z/t*	*P*
GA (week)		38.64 (37.71–39.71)	37.87 (36.14–38.00)	*Z* = -3.796	<0.001
BW (g)		3056.72(2700–3405)	2946.26(2510–3000)	*Z* = -1.962	0.050
Gender	male	60.50% (112)	57.80% (115)	0.300	0.584
	female	39.50% (73)	42.20% (83)		
Abnormal delivery		40.00% (74)	53.30% (106)	6.776	0.009
Single birth		96.80% (179)	87.90% (175)	10.349	0.001
Mother’s age(years old)		28.20(25.00–31.00)	29.62(29.62±4.32)	*t* = -3.071	0.002
GDM		9.20% (17)	26.80% (53)	19.786	<0.001
PIH		7.6% (14)	9.50% (19)	0.479	0.489
ICP		4.90% (9)	8.50% (17)	2.504	0.152
Intrauterine distress		8.10% (15)	11.60% (23)	1.280	0.258
MSAF		25.90% (48)	15.60% (31)	6.307	0.012
PROM		20.00% (37)	26.10% (52)	2.204	0.155
dystocia		3.80% (7)	1.50% (3)	1.958	0.162
Asphyxiation		12.40% (23)	12.60% (25)	0.001	0.969
Hypoglycemia		3.80% (7)	19.10% (38)	22.834	<0.001
Sepsis		1.10% (2)	20.60% (41)	37.900	<0.001
Severe jaundice		15.70% (29)	11.10% (22)	1.777	0.183
pH≤7.20		6.00% (7/116)	2.60% (5/196)	2.391	0.122

*Abbreviation: GA gestational age; BW birth weight; GDM gestational diabetes mellitus; PIH pregnancy-induced hypertension; ICP intrahepatic cholestasis of pregnancy; MSAF meconium-stained amniotic fluid; PROM premature rupture of membranes.

Compared with the pre-NNICU group, besides the smaller gestational age and lower birth weight, the risk factors during perinatal period, which considered be related to NE were more often found in post-NNICU group, including gestational diabetes mellitus (GDM), meconium-stained amniotic (MSAF), hypoglycemia and sepsis. Among these factors, however, only hypoglycemia and sepsis were statistically significant difference according to the multivariate analysis in this study. Patients with sepsis were more often found to have NE (*P* = 0.001, *OR* = 5.376), meanwhile, hypoglycemia (*P* = 0.001, *OR* = 22.737) and sepsis (*P* = 0.033, *OR* = 8.817) were more often found among patients with severe brain injury (Sarnat staging 3).

The clinical features were also shown in Table 2. The patients in the post-NNICU group had more severe clinical manifestations than those in the pre-NNICU group in general. A higher incidence of seizures was observed in the post-NNICU group (9.20% *vs* 22.10%, *P* = 0.001). Patients in the post-NNICU were more likely to have incomplete even absent primitive reflexes, especially sucking and moro reflexes (88.10% *vs* 96.50%, *P* = 0.002). Moreover, according to the Sarnat staging, more patients in the pre-NNICU were less than stage 1, whereas more patients in the post-NNICU group were evaluated as stage 2 (41.60% *vs* 58.80%, *P* = 0.001).

**Table 2:** Clinical features of the neonates.

		Pre-NNICU (n = 185)	Post-NNICU (n = 199)	*χ* ^*2*^ */Z*	*P*
Age of onset (days)		4.94(0–7.5)	5.11(0–8)	*Z* = -0.847	0.397
seizures		9.20% (17)	22.10% (44)	11.980	0.001
Abnormal level of consciousness		54.10% (100)	61.80% (123)	2.368	0.124
Hypotonia		20.50% (38)	18.60% (37)	0.231	0.631
Incomplete primitive reflexes		88.10% (163)	96.50% (192)	9.630	0.002
Hypothermia		0	0.50% (1)	-	-
Sarnat staging				*Z* = -4.148	<0.001
	Less than Stage1	50.80% (94)	31.20% (62)	15.355	<0.001
	Stage1	5.90% (11)	5.50% (11)	0.031	0.860
	Stage2	41.60% (77)	58.80% (117)	11.310	0.001
	Stage3	1.60% (3)	4.50% (9)	2.665	0.103

As summarized in Table 3, the overall frequency and the results neuromonitoring and neuroimaging showed some differences between the two groups. The aEEG was more often used in neuromonitoring in the post-NNICU group (*P*<0.001), most of the patients showed normal electrocortical background without significant difference between the two groups (67.90% *vs* 80.60%, *P* = 0.152). Compared with the pre-NNICU group, more patients had cranial ultrasound in the post-NNICU group, the difference was statistically significant (*P*<0.001). In addition, more patients in the pre-NNICU group had ventriculomegaly (29.30% *vs* 10.50%, *P* = 0.003) whereas more patients had cyst (7.30% *vs* 21.00%, *P* = 0.044) in the post-NNICU group. Furthermore, more patients had brain MRI in the post-NNICU group without significant difference (P = 0.061). The mean age at MRI in the pre-NNICU group were 5.87 days whereas 25.52 days in the post-NNICU group. Then, the results of brain MRI were recorded according to the National Institute of Child Health and Human Development Neonatal Research Network (NICHD), patients in the post-NNICU were more likely to have normal imaging in the first-time brain MRI (29.00% *vs* 49.50%, *P* = 0.009). Among the patients with brain MRI, only 4 patients had the second time MRI in the pre-NNICU group, and the follow-up rate of brain injury was higher in the post-NNICU group (5.80% *vs* 28.00%, *P*<0.001).

**Table 3:** Neuromonitoring and neuroimaging of the neonates.

		Pre-NNICU (n = 185)	Post-NNICU (n = 199)	*χ* ^*2*^ */Z*	*P*
aEEG		28	98	50.601	<0.001
normal		67.90% (19/28)	80.60% (79/98)	2.050	0.152
abnormal	mild	32.10% (9/28)	12.20% (12/98)	6.208	0.013
	moderate	0	7.10% (7/98)	2.118	0.146
	severe	0	0	-	-
Ultrasound		41	143	94.879	<0.001
normal		61.00% (25/41)	62.90% (90/143)	0.052	0.819
ventricular dilation		29.30% (12/41)	10.50% (15/143)	8.974	0.003
cyst		7.30% (3/41)	21.00(30/143)	4.041	0.044
IVH	I-II	2.40% (1/41)	3.50% (5/143)	0.113	0.737
	III-IV	0	2.10% (3/143)	0.874	0.350
MRI (first time)		69	93	3.500	0.061
Age at MRI (days)		5.87 (0–8)	25.52 (5.5–28.5)	*Z* = -6.849	<0.001
Stage 0		29.00% (20/69)	49.50% (46/93)	6.880	0.009
Stage 1	A	36.20% (25/69)	32.30% (30/93)	0.279	0.597
	B	21.70% (15/69)	11.80% (11/93)	2.888	0.089
Stage 2	A	5.80% (4/69)	2.20% (2/93)	1.477	0.224
	B	7.20% (5/69)	4.30% (4/93)	0.655	0.418
Stage 3		0	0	-	-
MRI (second time)		5.80% (4/69)	28.00% (26/93)	12.891	<0.001
Age at 2^nd^ time MRI (days)		159.5	111.96(45.75–133)	*Z* = -1.648	0.099
Stage 0		4	61.50% (16/26)		
Stage 1	A	0	34.60% (9/26)		
	B	0	3.80% (1/26)		
Stage 2	A	0	0		
	B	0	0		
Stage 3		0	0		
Improvement		100% (4/4)	92.30% (24/26)	0.330	0.566
MRI (third time)		0	3.22% (3/93)		

*Abbreviations: aEEG: amplitude-integrated electro encephalogram; IVH: intraventricular hemorrhage; MRI: magnetic resonance imaging.

The details of neuroprotective therapy were recorded in Table 4. There were more patients had liquid control during the acute phase in the post-NNICU group (40.00% *vs* 62.80%, *P*<0.001). Compared with the pre-NNICU group, the neuroprotective medicine without the evidence-based study were better controlled in the post-NNICU group (95.10% *vs* 16.60%, *P*<0.001). For the patients with neonatal asphyxia, therapeutic hypothermia was more used in the post-NNICU group (1.10% *vs* 8.00%, *P* = 0.001). Finally, patients in the post-NNICU group had shorter hospital stays (12.00 days *vs* 10.35 days, *P*<0.001) and most of them had good prognosis at discharge (75.70% *vs* 84.40%, *P* = 0.032).

**Table 4:** Neuroprotective therapy of the neonates.

		Pre-NNICU (n = 185)	Post-NNICU (n = 199)	*χ* ^*2*^ */Z*	*P*
Hospitalization (days)		12.00 (9–14)	10.35 (6–14)	*Z* = -4.035	<0.001
Therapeutic hypothermia (%)		1.10 (2)	8.00 (16)	10.392	0.001
Fluid restriction (%)		40.00 (74)	62.80 (125)	20.937	<0.001
Neuroprotective medicine		95.10 (176)	16.60% (33)	238.500	<0.001
Prognosis (discharge)	improvement	75.70% (140)	84.40% (168)	4.620	0.032
	deteriorate	24.30% (45)	15.60% (31)		

Compared with the post-NNICU group, more patients in the pre-NNICU group had the first-time assessment of neurodevelopment within 4 weeks afterbirth (94.10% vs 66.80%, P<0.001), while most of them had normal result in this assessment (66.70% *vs* 33.80%, *P*<0.001). During the 4 to 8 weeks after birth, the post-NNICU group had higher follow-up rate (60.90% vs 75.20%, P = 0.008) whereas the results were not statistically significant (P = 0.906). Meanwhile, the details for patient’s follow-up at 3 months after birth were also recorded. There was higher follow-up rate for the patients in the post-NNICU group (Table 5).

**Table 5:** The assessment of neurodevelopment for the neonates.

		Pre-NNICU (N = 185)	Post-NNICU (N = 199)	*χ* ^*2*^	*P*
1^st^ time (≤4 weeks)		94.10% (174/185)	66.80% (133/199)	44.310	<0.001
outcomes	normal	66.70% (116/174)	33.80% (45/133)		
	abnormal	33.30% (58/174)	66.20% (88/133)	32.580	<0.001
2^nd^ time (4–8 weeks)		60.90% (106/174)	75.20% (100/133)	6.952	0.008
outcomes	improvement	36.80% (39/106)	36.00% (36/100)		
	deteriorate	63.20% (67/106)	64.00% (64/100)	0.014	0.906
Follow-up at 3 months		7.00% (13/185)	25.60% (51/199)	23.884	<0.001
outcomes	normal	61.50% (8/13)	49.00% (25/51)		
	abnormal	38.50% (5/13)	51.00% (26/51)	0.650	0.420

## Discussion

Despite the great advances in neonatal intensive care management, the morbidity of neonatal encephalopathy (NE) maintains relatively high. On one hand, with the tremendous progress has been made in neonatology over the past years, especially the advances in life-support therapy, an increasing number of premature babies, extremely or very low birth weight infants, and the neonates with perinatal complications such as sepsis or hypoglycemia who are highly susceptible to NE are surviving [13–16]. On the other hand, elderly parturient women (especially more than 35 years old) and multiple pregnancies are also becoming more and more common in China due to the implementation of the two-child policy and the development of assisted reproduction technology, both of which are also reported to be associated with NE [17–21]. Therefore, specialized neurocritical care had been highly recommended to be provided to neonates deemed at risk of brain injury and poor outcome.

In this study, more newborns with the high risks of brain injury were admitted to Children’s Hospital of Chongqing Medical University (CHCMU) in recent years, especially neonates with sepsis or hypoglycemia which might be related to severe brain injury. Compared with the pre-NNICU group, more patients in post-NNICU group had incomplete even absent primitive reflexes or seizures, indicating more severe clinical manifestations, more than half of them (63.30%) had moderate to severe neonatal encephalopathy according to the Sarnat exam [22, 23]. This reflects that patient in the post-NNICU group had more serious primary disease, to improve the prognosis of them, the neurologic care should be broadly applied in the NNICU according to the current studies.

The concept of providing neurocritical care for neonates has gained momentum over the last decade, there have been several researches, guidelines and expert consensus published focusing on the standardized treatment for neonates with encephalopathy. According to these studies, the NNICU could provide specialized neurologic care by developing a multidisciplinary team trained in neurologic assessment, the implementation of neuroprotective strategies and brain monitoring.

According to current studies and guidelines, the clinical management for NE is primarily supportive care, with the additional neuroprotective therapy [1, 7, 12, 24]. Despite there have been several studies neuroprotective therapy for neonatal encephalopathy, such as the use of erythropoietin, Xenon, antioxidants and some neuroprotective medicine, none of them was proved to be effective on NE due to the lack of solid evidence-based reports [7, 24]. Therapeutic hypothermia is the only approach proven to decrease morbidity and mortality from neonates with HIE. There is a consensus among experts that therapeutic hypothermia should be more widely available, based upon the benefit and safety of hypothermia, without other effective approaches. Starting the hypothermia as soon as possible, especially within the first six hours after the insult is shown to improve outcomes in infants with moderate to severe encephalopathy [3]. According to a meta-analysis of seven randomized controlled trials of therapeutic hypothermia involving 1214 newborns with moderate to severe neonatal encephalopathy in 2012, therapeutic hypothermia reduced death or major neurodevelopmental disability and increased survival with a normal neurologic outcome at 18 months [25]. Aside from treatment with therapeutic hypothermia, the general supportive management are also important. The supportive care of neonatal encephalopathy includes respiratory, cardiovascular, neurological and metabolic condition et al [24]. Adequate ventilatory support is required to maintain oxygenation avoid hypoxia or over-ventilation. Neonates with NE in acute phase might complicate hypotension, shock, cardiomegaly, heart failure or ischemia, inotropic agents could be used to maintain blood pressure and adequate cerebral perfusion. However, systemic hypertension and fluid overload which might worsen encephaledema in acute stage of brain injury should be avoided. Fluid restriction is highly recommended for brain protection in the NNICU, especially for patients with HIE [24]. In this study, fluid restriction was better implemented after NNICU established. Moreover, therapeutic hypothermia was more often used in patients with HIE whereas the use of neuroprotective medicine without evidence-based study has been greatly dropped. In other words, after the establishment of the NNICU, neuroprotective strategies were more scientific and more in line with international guidelines.

Furthermore, supportive care in neurological is as important as therapeutic hypothermia in neuroprotective therapy. Patients with seizures might indicate a serious neurologic condition and poor prognosis. Phenobarbital was recommended in preventing and controlling seizures. However, detection of seizures by clinical observation is unreliable, distinguishing epileptic vs nonepileptic paroxysmal events that are detected at the bedside, even by the most experienced clinician, is accurate only approximately 50% of the time [26]. Neonates often have seizures without clinical correlate, especially after administration of seizure medications or in the setting of severe brain injury [27]. Among these neonates, continuous aEEG at the bedside in acute phase is highly recommended in order to detect subclinical seizures. In this study, after NNICU established, aEEG was more often used for neonates who were confirmed or suspected NE. Despite seizures were less found in the pre-NNICU group, there was a higher incidence of abnormal aEEG monitoring, that is to say, some of the patients only had EEG confirmed seizures rather than clinically apparent. The results of aEEG monitoring might provide the evidence for anticonvulsant drugs. The previous studies have reported a significant improvement in electrographic seizure detection, lower phenobarbital burden, less anticonvulsant therapy at discharge and shorter length of stay associated with a continuous EEG in the NNICU. Prolonged aEEG monitoring might help improve prognosis.

As a serious neurological dysfunction syndrome, neonatal encephalopathy might cause severe neurological disabilities, so long-term, regular follow-up and evaluation are critical. The assessment of neurodevelopment combined neuroimaging is usually used in patients with NE, especially cranial MRI. In previous studies, the brain MRI was reported to have some predictive value in neurodevelopment, the imaging features in brain MRI might be considered as potential biomarkers of long-term outcome, whereas it is associated with the age of examination [28]. In this study, the average for first MRI was 5.87 days in pre-NNICU group whereas 25.52 days in post-NNICU group. The first week of birth for most of patients is the acute phase of disease, most of them have critical conditions, the result of brain MRI in acute stage of disease did not have significant effect on neuroprotective therapy. Moreover, the brain MRI requires sedation and takes at least half an hour, it is not suitable and safe for a critical neonate. On the other hand, a published systematic review also showed that late MRI (8–30 days) had higher sensitivity and MRI within 2 weeks of birth correctly predict neurological outcomes at 18 months of age [29, 30]. Therefore, after NNICU established, standardized neuroprotective strategies were more often considered, imaging evaluations were performed in relatively safe and meaningful conditions, which might help to improve clinical quality.

In addition, the assessment of neurodevelopment might show the long-term neurological development of the patients, especially the result of assessment in 3 months of age, which is considered to be associated with neurodevelopment in 18 months age [31]. In the pre-NNICU group, most of the patients were assessed normal neurological development whereas more subnormal conditions were found during follow-up, more than 90% of patients lost to follow-up in this observation period. On the contrary, the assessment of neurodevelopment was gradually improved during follow-up in the post-NNICU group, although most of them had abnormal result in the first assessment, moreover, the follow-up rate in 3 months of age was much higher than before. During the previous observation period, patients were assessed by NBNA or DQ, while TIMP was widely used in the latter observation period. The NBNA is mostly used in china since 1989, which is simple and convenient without a better repeatability and accuracy. The TIMP was established by Campbell et al. in 2005, it is a 42-item assessment of postural and selective control needed for function in early infancy, which is used around the world [32]. The TIMP has better repeatability and accuracy [33]. Therefore, after NNICU established, patients had better follow-up compliance in the assessment of neurodevelopment, the result of assessment was more objective and instructive.

In general, under the guidance of evidence-based medicine, patients in the post-NNICU group had more serious primary disease but better prognosis while discharge and similar short-term prognosis at 3 months old during follow-up, indicating that neonates with encephalopathy might benefit from the NNICU establishment, which might improve the neurodevelopment outcomes of NE newborns.

## Conclusions

The NNICU focused on the neonatal neurocritical care for the babies susceptible to NE with the guidance of evidence-based medicine, the establishment of NNICU is gradually improving and standardizing the neuroprotective therapy and clinical follow-up to improve neurodevelopmental prognosis of the NE patients in CHCMU.
